# Class 1 Integrons and Antibiotic Resistance of Clinical *Acinetobactercalcoaceticus*–*baumannii* Complex in Poznań, Poland

**DOI:** 10.1007/s00284-014-0581-0

**Published:** 2014-04-17

**Authors:** Ryszard Koczura, Beata Przyszlakowska, Joanna Mokracka, Adam Kaznowski

**Affiliations:** Department of Microbiology, Faculty of Biology, Adam Mickiewicz University in Poznań, ul Umultowska 89, 61-614 Poznan, Poland

## Abstract

Sixty-three clinical isolates of *Acinetobacter calcoaceticus*–*baumannii* complex were analyzed for the presence of integrons and antimicrobial resistance. Class 1 integrons were detected in 40 (63.5 %) isolates. None of them had class 2 or class 3 integrons. The majority of the integrons contained *aacC1*–*orfA*–*orfB*–*aadA1* gene cassette array. The presence of integrons was associated with the increased frequency of resistance to 12 of 15 antimicrobials tested, multi-drug resistance phenotype, and the overall resistance ranges of the strains.

**Electronic supplementary material:**

The online version of this article (doi:10.1007/s00284-014-0581-0) contains supplementary material, which is available to authorized users.

## Introduction

*Acinetobacter* spp. are Gram-negative, strictly aerobic bacteria ubiquitous in the environment and in clinical settings. The genus comprises 33 named and unnamed genospecies. The *Acinetobacter calcoaceticus*–*baumannii* complex includes four genospecies, i.e., *A.* *calcoaceticus*, *A.* *baumannii*, and hybridization groups 3 and 13 TU that cannot be differentiated by phenotypic tests [8]. Members of the *A.* *calcoaceticus*–*baumannii* complex account for about 75 % of *Acinetobacter* spp. isolated from clinical specimens [14] and are etiological agents of a wide spectrum of nosocomial infections, including blood and urinary tract infections, and pneumonia [22, 34]. Many infections can be severe with mortality ranging from 26 to 70 % [28].

Antimicrobial resistance of *A.* *calcoaceticus*–*baumannii* complex isolates has been increasing continuously [21]. Clinical isolates are frequently resistant to most antimicrobials commonly used against Gram-negative pathogens. In recent years, a growing number of carbapenem-resistant strains have been reported in Europe [13].

One of the genetic elements involved in the spread of resistance among bacteria are integrons. They are genetic elements responsible for integration and rearrangements of resistance determinants called gene cassettes [32]. Integrons consist of an integrase gene, a primary recombination site called *attI*, and a promoter P_C_ that directs transcription of the integrated genes. Several classes of integrons are distinguished upon the sequences of the integrase genes. The most common integrons belong to class 1 and play an important role in the emergence and spread of resistance genes [2, 31]. Bacterial strains harboring class 1 integrons are usually multiresistant, i.e., resistant to antimicrobials of at least three different classes. This has been proven for many species of the *Enterobacteriaceae* family, including *Escherichia coli*, *Enterobacter hormaechei*, *E. aerogenes*, *Citrobacter freundii*, *Klebsiella pneumoniae*, *K. oxytoca*, *Serratia marcescens*, and *Proteus mirabilis* [17, 24, 25].

The aim of this study was to investigate the presence of integrons in clinical isolates of the *A.* *calcoaceticus*–*baumannii* complex, with a focus on antimicrobial resistance of the isolates and gene cassette content of the integrons.

## Materials and Methods

### Bacterial Isolates

Sixty-three *A. calcoaceticus*–*baumannii* complex isolates were cultured from specimens taken from inpatients at a hospital in Poznań, Poland, and identified with API 20NE (bioMérieux). The isolates were collected between 2000 and 2010. The specimens included urine, blood, wound swabs, and tracheobronchial aspirates.

### BOX–PCR Typing

The genetic similarity of the isolates was determined by BOX–PCR typing with BOX A1R primer according to Versalovic et al. [37]. The PCR products were separated in agarose gel, stained with ethidium bromide and digitalized with Bio-Print v. 99 gel documentation system (Vilbert-Lourmat). The electrophoretic patterns were analyzed by GelCompar II 3.5 software (Applied Maths) with internal and external normalization. Optimization and band position tolerance were set at 1 %. Similarity between fingerprints was calculated with the Dice coefficient. Clustering was carried out by the unweighted pair–group method with average linkages (UPGMA).

### Detection of Integrase Genes

Multiplex PCR assay for the detection of *intI1*, *intI2*, and *intI3* integrase genes characteristics for class 1, class 2, and class 3 integrons, respectively, was performed according to Dillon et al. [4]. Amplification involved an initial denaturation (94 °C, 5 min) followed by 30 cycles of denaturation (94 °C, 1 min), annealing (59 °C, 1 min), and extension (72 °C, 1 min) with a final extension step (72 °C, 5 min).

### Analysis of the Variable Regions of Class 1 Integrons

The variable regions of class 1 integrons were PCR-amplified with primers 5′-CS and 3′-CS recommended by Lévesque et al. [18]. The PCR reaction was conducted as follows: initial denaturation 94 °C, 5 min, and 30 cycles of 94 °C 1 min., 55 °C 1 min., 72 °C 5 min., and final elongation 72 °C 8 min. The amplicons were purified with ExoSAP-IT (Affymetrix) and sequenced in a 3130xl Genetic Analyzer (Applied Biosystems). The sequences were assembled with DNA Baser (HeracleSoftware) and aligned with available GenBank data using nucleotide BLAST (Basic Local Alignment Search Tool).

All PCR reactions were carried out in a C1000 Thermal Cycler (BioRad) with primers synthesized by Oligo.pl and HiFi *Taq* polymerase provided by Novazym. The PCR products were separated in 1.5 % agarose gel (Novazym). Molecular weight of PCR products was determined with GelCompar II 3.5. The experiments were performed in duplicate.

### Detection of Integrons in Plasmids

Plasmid DNA was isolated using Plasmid Mini AX (A&A Biotechnology) kit following manufacturer’s instruction. Plasmids were separated in a 0.7 % agarose gel, and molecular size of the bands was determined with GelCompar II 3.5; *E. coli* V-517 plasmids were used as molecular size markers. All plasmids were cut off the gel and eluted by Gel-Out kit (A&A Biotechnology). Integrons in plasmids were detected as described above.

### Antibiotic Susceptibility Testing

Antibiotic susceptibility of the isolates was determined by standard disk diffusion method on Mueller-Hinton agar (Oxoid) according to the guidelines and breakpoints of Clinical and Laboratory Standards Institute [3]. The isolates were tested for susceptibility to 15 antibiotics representing seven classes: amikacin (30 µg), ampicillin/sulbactam (20 µg), cefepime (30 µg) cefotaxime (30 µg), ceftazidime (30 µg), chloramphenicol (30 µg), ciprofloxacine (5 µg), gentamicin (10 µg), imipenem (10 µg), piperacillin (100 µg), piperacillin/tazobactam (110 µg), sulfamethoxazole/trimethoprim (co-trimoxazole) (25 µg), tetracycline (30 µg), and tobramycin (10 µg). Resistance to netilmicin (10 µg) was determined according to European Committee on Antimicrobial Susceptibility Testing breakpoints v. 3.0 [5] All antibiotic disks were provided by Oxoid. The quality control strain was *E. coli* ATCC 25922.

### Statistical Analysis

The frequencies of resistance to particular antimicrobials in integron-positive and -negative isolates were compared with Fisher’s exact test. The differences in antimicrobial resistance ranges, expressed as the number of antimicrobial classes to which the isolates were resistant, were compared with Mann–Whitney *U* test. *P* < 0.05 was considered to indicate statistical significance. Calculations were performed with Statistica 10 software (StatSoft).

## Results

We analyzed 63 clinical isolates of the *A. calcoaceticus*–*baumannii* complex for the presence of integrons and their antimicrobial resistance. In order to avoid analyzing repetitive clones, we carried out BOX–PCR typing. Although it indicated the presence of two clusters consisting of three and two isolates with identical band patterns (Supplementary Fig. S1), subsequent analyses showed differences between their resistance profiles, which may be explained by localization of some antimicrobial resistance determinants on plasmids.

Screening for the presence of integron integrase genes showed the presence of class 1 integrase gene in 40 (63.5 %) clinical isolates of *A. calcoaceticus*–*baumannii* complex. None of the isolates had class 2 or class 3 integrons. Class 1 integron-harboring strains carried several plasmids (Supplementary Fig. S2); however, PCR analysis of the plasmids showed the integrons were localized only in a ~60-kbp plasmid.

The amplification of the variable regions of class 1 integrons yielded a 2.5-kbp amplicon in 34 isolates and a 1.9-kbp PCR product in one isolate. We did not obtain an amplicon of the variable region in five (12.5 %) integron-positive isolates. Sequence analysis showed the presence of gene cassettes conferring resistance to aminoglycosides (*aadA1*, *aadA2*, and *aacC1*), trimethoprim (*dfrA12*), as well as genes of unknown function. All of the 2.5-kbp amplicons consisted of *aacC1*–*orfA*–*orfB*–*aadA1* gene cassette arrays, whereas the 1.9-kbp amplicon contained a *dfrA12*–*orfF*–*aadA2* array.

The frequencies of antimicrobial resistance of integron-positive and integron-negative *A. calcoaceticus*–*baumannii* complex isolates are presented in Table 1. Among the integron-positive strains, the highest resistance frequency was noted for cefepime, cefotaxime, and tetracycline, and the lowest to netilmicin. Strains without integrons were also most frequently resistant to cefepime and least frequently resistant to netilmicin. For most antimicrobials, the frequency of resistance was higher in the group of strains that harbored class 1 integrons. No statistically significant differences were found for imipenem, netilmicin, and tobramycin.

**Table 1:** Antimicrobial resistance of integron-positive and integron-negative isolates of *Acinetobacter* *calcoaceticus*–*baumannii* complex

Antimicrobial	*intI*(+) *n* = 40 (%)	*intI*(−) *n* = 23 (%)	*P* value
Amikacin	34 (85.0)	9 (39.1)	<0.001^a^
Gentamicin	36 (90.0)	10 (43.5)	<0.001
Netilmicin	6 (15.0)	3 (13.0)	1.000
Tobramycin	14 (90.2)	5 (41.7)	0.394
Ampicillin/sulbactam	33 (80.5)	12 (52.2)	0.019
Piperacillin	38 (95.0)	14 (60.9)	0.001
Piperacillin/tazobactam	36 (90.0)	12 (52.2)	0.001
Ticarcillin	37 (92.5)	12 (52.2)	<0.001
Cefepime	39 (97.5)	18 (78.3)	0.021
Cefotaxime	39 (97.5)	16 (69.6)	0.003
Ceftazidime	37 (92.5)	12 (52.2)	<0.001
Imipenem	18 (45.0)	8 (34.8)	0.596
Ciprofloxacin	36 (90.0)	8 (34.8)	<0.001
Tetracycline	39 (97.5)	15 (65.2)	0.001
Sulfamethoxazole/trimethoprim	37 (92.5)	11 (47.8)	<0.001

^a^ *P* value calculated in Fisher’s exact test

**Fig. 1:**
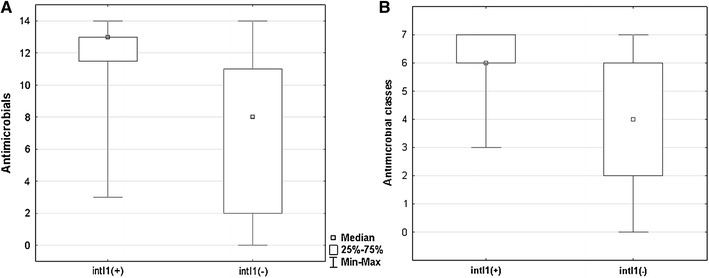
Box plots of the number of antimicrobials (**a**) and antimicrobial classes (**b**) to which integron-positive and integron-negative *A. calcoaceticus*–*baumannii* complex isolates were resistant

The integron-bearing strains were resistant to 3–14 of the antimicrobials belonging to 3–7 classes, i.e., all of them were multi-drug resistant (Fig. 1). The integron-negative isolates were resistant to 0–14 drugs of 0–7 classes and fifteen of them (65.2 %) were multi-drug resistant (MDR). The difference in the MDR frequency between strains with and without integrons was statistically significant (*P* < 0.001, Fisher’s exact test). The differences in resistance ranges, defined either as the number of antimicrobials or antimicrobial classes the strains were resistant to, between integron-positive and -negative strains were statistically significant (*P* < 0.001, *U* Mann–Whitney test).

## Discussion

*Acinetobacter calcoaceticus*–*baumannii* complex has become an emerging etiological agent of nosocomial infections [11]. Surveys have shown increasing resistance among clinical isolates and emergence of MDR strains, likely due to overuse of antimicrobials, which limits therapeutic options. One of the most effective drugs against *A. calcoaceticus*–*baumannii* complex are carbapenems. In England, approximately 15 % of isolates were resistant to imipenem [35]; however, in Spain, higher frequency of resistance to imipenem has been reported, ranging from 21 to 80 %, depending on location and the time of study [36]. In Asia, resistance to imipenem among *A. calcoaceticus*–*baumannii* complex has been also reported, with frequency reaching 36 % in Nepal [23] and 59 % in Taiwan [15]. In our study, 41 % of isolates were resistant to imipenem, whereas Bogiel et al. [1] have reported 27.6–31.0 % of imipenem-resistant strains isolated from a hospital in Bydgoszcz, Poland, and Wróblewska et al. [38] have found 13.6 % of isolates intermediately susceptible or resistant to that drug in a hospital in Warsaw.

Genes determining antimicrobial resistance in Gram-negative bacteria are often located within integrons [2]. We found class 1 integrons in 63.5 % of *A.* *calcoaceticus*–*baumannii* complex isolates, which is similar to the frequency of integron-positive strains among *A. baumannii* isolated in Iran—74.0 % [28], Taiwan—71.4 % [9], and South Africa—65.6 % [30]. Much higher percentage (80.4 %) was observed among *A. calcoaceticus*–*baumannii* complex strains originated from a hospital in China [19]. Class 1 integrons in *Acinetobacter* spp. have been localized in different plasmids, ranging from 60 kbp [12, this study] to 120 kbp [20], but they can occur in the chromosome as well [20]. We did not detect the presence of class 2 and class 3 integrons. Class 2 integrons are found with low frequency in various Gram-negative bacteria [25, 26, 39]. In *Acinetobacter* spp., class 2 integron has been found only in limited number of strains, originated from a hospital in Brazil [7], from hospitals in Iran [10, 29, 33], and from wastewater in China [39]. Class 3 integrons have not been detected in *Acinetobacter* spp. so far.

There was little diversity in the genetic content of the class 1 integrons. We identified only two gene cassette arrays: *aacC1*–*orfA*–*orfB*–*aadA1* and *dfrA12*–*orfF*–*aadA2*, conferring resistance to aminoglycosides and trimethoprim, and all of the integron-positive isolates displayed resistance to those antimicrobials. As much as 12.5 % of the isolates did not yield a PCR product of the integron’s variable region, which can be explained by altered sequence or the lack of *sul1* gene in the 3′ conserved region of class 1 integrons [16]. The same gene cassette arrays have been found in *A. baumannii* in Taiwan [9]. An *A. calcoaceticus*–*baumannii* complex strain isolated from Poland contained a plasmid-located integron with *bla*_VIM-2-_ *aacA4* gene array [6].

The genes of class 1 integrons of *A. calcoaceticus*–*baumannii* complex coded only for resistance to aminoglycosides and trimethoprim. Nevertheless, resistance to penicillins, cephalosporins, ciprofloxacin, tetracycline, and sulfamethoxazole/trimethoprim was associated with the presence of an integron as well, and all strains with integrons were multi-drug resistant. This phenomenon appears to be common in integron-bearing bacteria [10, 24, 25], likely to the presence of numerous resistance determinants in one genetic element, e.g., a plasmid. This may also explain higher resistance ranges of integron-positive *A. calcoaceticus*–*baumannii* complex strains in this study, as the integrons were located in a 60-kbp plasmid. It has been proved that multidrug resistance phenotype in bacteria, especially those of nosocomial origin, can be spread through plasmids containing class 1 integrons that can be readily transferred between different species [27].

In conclusion, the presence of class 1 integrons among clinical isolates of *A.* *calcoaceticus*–*baumannii* complex appears to be common in Poland. Since they are associated with MDR phenotype, the presence of class 1 integrons may be used as a marker of multi-drug resistance.

## Electronic supplementary material

Below is the link to the electronic supplementary material.

Supplementary Fig. S1 Dendrogram showing antimicrobial resistance profiles and genetic relatedness of 63 *A. calcoaceticus*–*baumannii* complex isolates determined by BOX–PCR analysis with Dice coefficient and UPGMA clustering method. Antimicrobial symbols: *AMK* amikacin, *GEN* gentamicin, *NET* netilmicin, *TOB* tobramycin, *SAM* ampicillin/sulbactam, *PIP* piperacillin, *TZP* piperacillin/tazobactam, *TIC* ticarcillin, *FEP* cefepime, *CTX* cefotaxime, *CAZ* ceftazidime, *IMP* imipenem, *CIP* ciprofloxacin, *TET* tetracycline, *SXT* sulfamethoxazole/trimethoprim (PDF 333 kb)

Supplementary Fig. S2 Agarose gel electrophoresis of plasmids extracted from class 1 integron-bearing *A. calcoaceticus*–*baumannii* complex isolates. Line 1, *E. coli* V-517 (plasmid sizes in kpb given on the *left*); lines 2–9, *A. calcoaceticus*–*baumannii* complex isolates. Plasmids containing class 1 integrons indicated by *white arrow* (PDF 254 kb)
