# Supplementary material for: Class 1 Integrons and Antibiotic Resistance of Clinical Acinetobactercalcoaceticus–baumannii Complex in Poznań, Poland
Source: Curr Microbiol. 2014 Apr 17;69(3):258–62. doi: 10.1007/s00284-014-0581-0 (PMC4113676; doi:10.1007/s00284-014-0581-0)
Supplement: Supplementary file 1 — Dendrogram showing antimicrobial resistance profiles and genetic relatedness of 63 A. calcoaceticus–baumannii complex isolates determined by BOX–PCR analysis with Dice coefficient and UPGMA clustering method. Antimicrobial symbols: AMK amikacin, GEN gentamicin, NET netilmicin, TOB tobramycin, SAM ampicillin/sulbactam, PIP piperacillin, TZP piperacillin/tazobactam, TIC ticarcillin, FEP cefepime, CTX cefotaxime, CAZ ceftazidime, IMP imipenem, CIP ciprofloxacin, TET tetracycline, SXT sulfamethoxazole/trimethoprim (PDF 333 kb) [file 284_2014_581_MOESM1_ESM.pdf]

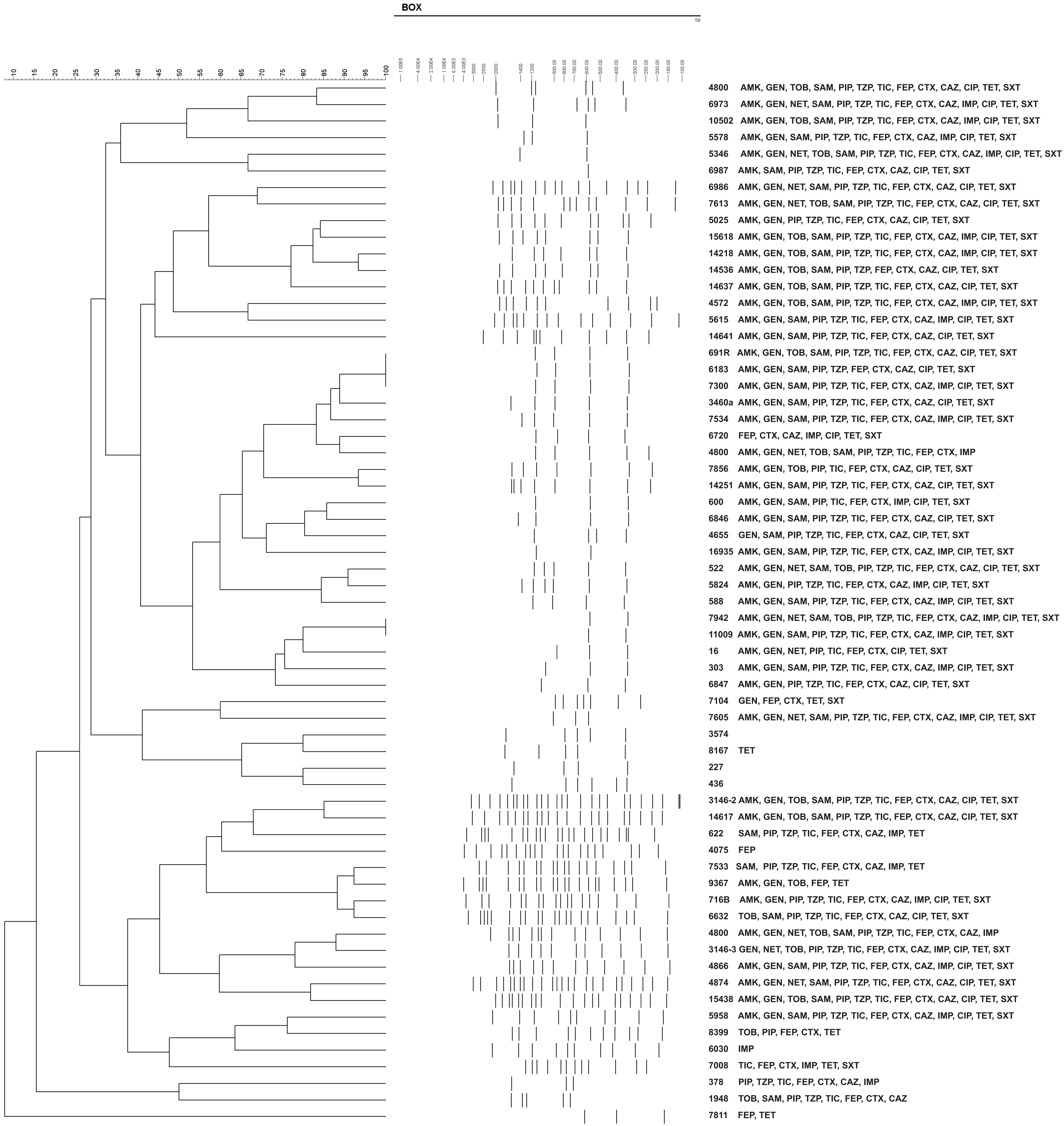

BOX
bp
10 15 20 25 30 35 40 45 50 55 60 65 70 75 80 85 90 95 100
1.00E5 4.00E4 2.00E4 1.00E4 6.00E3 4.00E3 3000 2500 2000 1400 1200 900.00 800.00 700.00 600.00 500.00 400.00 300.00 250.00 200.00 150.00 100.00
4800 AMK, GEN, TOB, SAM, PIP, TZP, TIC, FEP, CTX, CAZ, CIP, TET, SXT
6973 AMK, GEN, NET, SAM, PIP, TZP, TIC, FEP, CTX, CAZ, IMP, CIP, TET, SXT
10502 AMK, GEN, TOB, SAM, PIP, TZP, TIC, FEP, CTX, CAZ, IMP, CIP, TET, SXT
5578 AMK, GEN, SAM, PIP, TZP, TIC, FEP, CTX, CAZ, IMP, CIP, TET, SXT
5346 AMK, GEN, NET, TOB, SAM, PIP, TZP, TIC, FEP, CTX, CAZ, IMP, CIP, TET, SXT
6987 AMK, SAM, PIP, TZP, TIC, FEP, CTX, CAZ, CIP, TET, SXT
6986 AMK, GEN, NET, SAM, PIP, TZP, TIC, FEP, CTX, CAZ, CIP, TET, SXT
7613 AMK, GEN, NET, TOB, SAM, PIP, TZP, TIC, FEP, CTX, CAZ, CIP, TET, SXT
5025 AMK, GEN, PIP, TZP, TIC, FEP, CTX, CAZ, CIP, TET, SXT
15618 AMK, GEN, TOB, SAM, PIP, TZP, TIC, FEP, CTX, CAZ, IMP, CIP, TET, SXT
14218 AMK, GEN, TOB, SAM, PIP, TZP, TIC, FEP, CTX, CAZ, IMP, CIP, TET, SXT
14536 AMK, GEN, TOB, SAM, PIP, TZP, FEP, CTX, CAZ, CIP, TET, SXT
14637 AMK, GEN, TOB, SAM, PIP, TZP, TIC, FEP, CTX, CAZ, CIP, TET, SXT
4572 AMK, GEN, TOB, SAM, PIP, TZP, TIC, FEP, CTX, CAZ, IMP, CIP, TET, SXT
5615 AMK, GEN, SAM, PIP, TZP, TIC, FEP, CTX, CAZ, IMP, CIP, TET, SXT
14641 AMK, GEN, SAM, PIP, TZP, TIC, FEP, CTX, CAZ, CIP, TET, SXT
691R AMK, GEN, TOB, SAM, PIP, TZP, TIC, FEP, CTX, CAZ, CIP, TET, SXT
6183 AMK, GEN, SAM, PIP, TZP, FEP, CTX, CAZ, CIP, TET, SXT
7300 AMK, GEN, SAM, PIP, TZP, TIC, FEP, CTX, CAZ, IMP, CIP, TET, SXT
3460a AMK, GEN, SAM, PIP, TZP, TIC, FEP, CTX, CAZ, CIP, TET, SXT
7534 AMK, GEN, SAM, PIP, TZP, TIC, FEP, CTX, CAZ, IMP, CIP, TET, SXT
6720 FEP, CTX, CAZ, IMP, CIP, TET, SXT
4800 AMK, GEN, NET, TOB, SAM, PIP, TZP, TIC, FEP, CTX, IMP
7856 AMK, GEN, TOB, PIP, TIC, FEP, CTX, CAZ, CIP, TET, SXT
14251 AMK, GEN, SAM, PIP, TZP, TIC, FEP, CTX, CAZ, CIP, TET, SXT
600 AMK, GEN, SAM, PIP, TIC, FEP, CTX, IMP, CIP, TET, SXT
6846 AMK, GEN, SAM, PIP, TZP, TIC, FEP, CTX, CAZ, CIP, TET, SXT
4655 GEN, SAM, PIP, TZP, TIC, FEP, CTX, CAZ, CIP, TET, SXT
16935 AMK, GEN, SAM, PIP, TZP, TIC, FEP, CTX, CAZ, IMP, CIP, TET, SXT
522 AMK, GEN, NET, SAM, TOB, PIP, TZP, TIC, FEP, CTX, CAZ, CIP, TET, SXT
5824 AMK, GEN, PIP, TZP, TIC, FEP, CTX, CAZ, IMP, CIP, TET, SXT
588 AMK, GEN, SAM, PIP, TZP, TIC, FEP, CTX, CAZ, IMP, CIP, TET, SXT
7942 AMK, GEN, NET, SAM, TOB, PIP, TZP, TIC, FEP, CTX, CAZ, IMP, CIP, TET, SXT
11009 AMK, GEN, SAM, PIP, TZP, TIC, FEP, CTX, CAZ, IMP, CIP, TET, SXT
16 AMK, GEN, NET, PIP, TIC, FEP, CTX, CIP, TET, SXT
303 AMK, GEN, SAM, PIP, TZP, TIC, FEP, CTX, CAZ, IMP, CIP, TET, SXT
6847 AMK, GEN, PIP, TZP, TIC, FEP, CTX, CAZ, CIP, TET, SXT
7104 GEN, FEP, CTX, TET, SXT
7605 AMK, GEN, NET, SAM, PIP, TZP, TIC, FEP, CTX, CAZ, IMP, CIP, TET, SXT
3574
8167 TET
227
436
3146-2 AMK, GEN, TOB, SAM, PIP, TZP, TIC, FEP, CTX, CAZ, CIP, TET, SXT
14617 AMK, GEN, TOB, SAM, PIP, TZP, TIC, FEP, CTX, CAZ, CIP, TET, SXT
622 SAM, PIP, TZP, TIC, FEP, CTX, CAZ, IMP, TET
4075 FEP
7533 SAM, PIP, TZP, TIC, FEP, CTX, CAZ, IMP, TET
9367 AMK, GEN, TOB, FEP, TET
716B AMK, GEN, PIP, TZP, TIC, FEP, CTX, CAZ, IMP, CIP, TET, SXT
6632 TOB, SAM, PIP, TZP, TIC, FEP, CTX, CAZ, CIP, TET, SXT
4800 AMK, GEN, NET, TOB, SAM, PIP, TZP, TIC, FEP, CTX, CAZ, IMP
3146-3 GEN, NET, TOB, PIP, TZP, TIC, FEP, CTX, CAZ, IMP, CIP, TET, SXT
4866 AMK, GEN, SAM, PIP, TZP, TIC, FEP, CTX, CAZ, IMP, CIP, TET, SXT
4874 AMK, GEN, NET, SAM, PIP, TZP, TIC, FEP, CTX, CAZ, CIP, TET, SXT
15438 AMK, GEN, TOB, SAM, PIP, TZP, TIC, FEP, CTX, CAZ, CIP, TET, SXT
5958 AMK, GEN, SAM, PIP, TZP, TIC, FEP, CTX, CAZ, IMP, CIP, TET, SXT
8399 TOB, PIP, FEP, CTX, TET
6030 IMP
7008 TIC, FEP, CTX, IMP, TET, SXT
378 PIP, TZP, TIC, FEP, CTX, CAZ, IMP
1948 TOB, SAM, PIP, TZP, TIC, FEP, CTX, CAZ
7811 FEP, TET
