# Supplementary figures and images for: Class 1 Integrons and Antibiotic Resistance of Clinical Acinetobactercalcoaceticus–baumannii Complex in Poznań, Poland
Source: Curr Microbiol. 2014 Apr 17;69(3):258–62. doi: 10.1007/s00284-014-0581-0 (PMC4113676; doi:10.1007/s00284-014-0581-0)

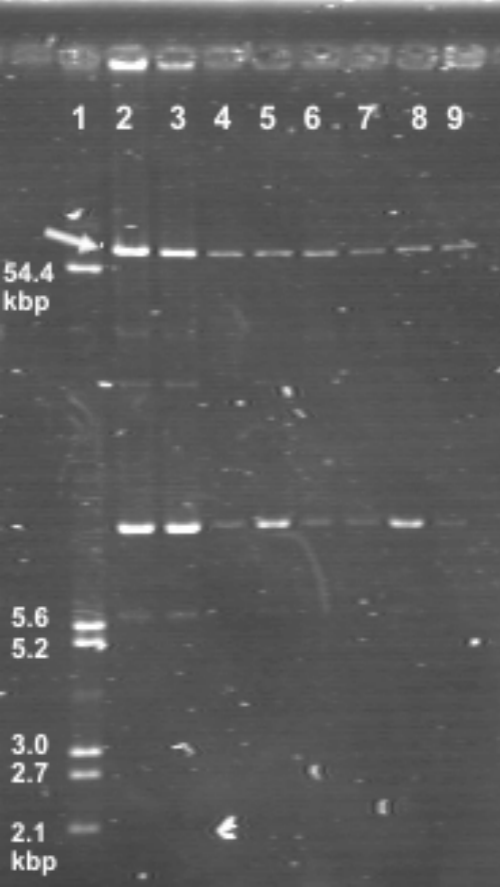

Supplement: Supplementary file 2 — Agarose gel electrophoresis of plasmids extracted from class 1 integron-bearing A. calcoaceticus–baumannii complex isolates. Line 1, E. coli V-517 (plasmid sizes in kpb given on the left); lines 2–9, A. calcoaceticus–baumannii complex isolates. Plasmids containing class 1 integrons indicated by white arrow (PDF 254 kb) [file 284_2014_581_MOESM2_ESM.pdf]
